# Combination of Low-Dose Gemcitabine and PD-1 Inhibitors for Treatment in Patients With Advanced Malignancies

**DOI:** 10.3389/fimmu.2022.882172

**Published:** 2022-07-13

**Authors:** Hao Huang, Ling Peng, Bicheng Zhang, Brian G. Till, Yonghao Yang, Xiaojie Zhang, Lingdi Zhao, Xiaomin Fu, Tiepeng Li, Lu Han, Peng Qin, Lin Chen, Xiang Yan, Yang Liu, Wenkang Wang, Zhenlong Ye, Hongle Li, Quanli Gao, Zibing Wang

**Affiliations:** ^1^Department of Immunotherapy, the Affiliated Cancer Hospital of Zhengzhou University & Henan Cancer Hospital, Zhengzhou, China; ^2^Department of Respiratory Disease, Zhejiang Provincial People’s Hospital, Hangzhou, China; ^3^Department of Oncology, Renmin Hospital of Wuhan University, Wuhan, China; ^4^Clinical Research Division, Fred Hutchinson Cancer Research Center, Seattle, WA, United States; ^5^Guangzhou Medical University-Guangzhou Institute of Biomedicine and Health GZMU-GIBH Joint School of Life Sciences, Guangzhou Medical University, Guangzhou, China; ^6^Medical Oncology Department, Chinese People's Liberation Army PLA General Hospital, Beijing, China; ^7^Department of Radiotherapy, the Affiliated Cancer Hospital of Zhengzhou University, Henan Cancer Hospital, Zhengzhou, China; ^8^Department of Breast Surgery, the First Affiliated Hospital of Zhengzhou University, Zhengzhou, China; ^9^Mengchao Cancer Hospital, Shanghai University, Shanghai, China; ^10^Department of Immune Cell Research, Shanghai Engineering Research Center for Cell Therapy, Shanghai, China; ^11^School of Pharmacy, Binzhou Medical University, Binzhou, China; ^12^Molecular Pathology Department, the Affiliated Cancer Hospital of Zhengzhou University and Henan Cancer Hospital, Zhengzhou, China

**Keywords:** combination immunotherapy, PD-1 inhibitors, low-dose chemotherapy, gemcitabine, malignancy

## Abstract

**Purpose:**

This study determined the efficacy of low-dose gemcitabine combined with programmed death-1 (PD-1) inhibitors for treating multiple malignancies, providing a cost-effective and safe treatment option.

**Study Design:**

This study included 61 patients with advanced solid tumors treated with low-dose gemcitabine combined with PD-1 inhibitors at the Henan Cancer Hospital between January 2018 and February 2022. We retrospectively reviewed medical records to evaluate several clinical factors, including progression-free survival (PFS), overall survival (OS), adverse effects (AEs), and objective response to treatment.

**Results:**

Sixty-one patients received treatment with low-dose gemcitabine combined with PD-1 inhibitors. The objective response rate (ORR) was 29.5% and the disease control rate (DCR) was 62.3%. The median PFS was 4.3 months (95% confidence interval, 2.3 to 6.3 months) and the median OS was 15.0 months (95% confidence interval, 8.8 to 21.2 months). Hematological toxicity, mainly leukopenia or thrombocytopenia, was the most common AE, with any-grade and grade 3/4 hematological toxicity reported in 60.7 and 13.1% of patients, respectively.

**Conclusions:**

Low-dose gemcitabine combined with PD-1 inhibitors may offer a novel treatment option for patients with advanced malignancies.

## Introduction

The advent of immune checkpoint inhibitors (ICIs) has driven the progress of tumor therapy, transformed the treatment landscape of multiple tumor types and provided clinicians with new therapeutic strategies (1, 2). However, since the overall response rate of ICI therapy is around 20%, only a small proportion of patients benefit from this treatment (3). Recent findings demonstrate that some chemotherapeutics given using specific administration schedules display positive immunological effects that contribute to tumor eradication (4–6). Therefore, there is a growing interest in combining ICIs with chemotherapy to enhance the efficacy of immunotherapy.

In established tumor models, gemcitabine induces tumor cell apoptosis and thereby elicits antitumor immunity by increasing the amount of antigen cross-presentation (7–10). Gemcitabine also enhances CD8^+^ T cell and natural killer (NK) cell-mediated anti-tumor immunity through depletion of myeloid-derived suppressor cells and regulatory T cells (11). Some preclinical data provide a rationale for combining gemcitabine with ICIs (12, 13); however, the clinical efficacy of gemcitabine combined with ICIs for treating solid tumors is not as expected. For example, in the first-line treatment of advanced non-small-cell lung cancer, progression-free survival (PFS) and overall survival (OS) in patients treated with gemcitabine combined with nivolumab did not increase compared with single drug use (14). In pancreatic ductal adenocarcinoma, gemcitabine plus ipilimumab achieves a similar objective response rate (ORR) to gemcitabine monotherapy (15). The same results were reported in metastatic disease or locoregional nasopharyngeal carcinoma patients treated with camrelizumab (SHR-1210) along with gemcitabine (16).

Multiple factors may contribute to the lack of a significant increase in the efficacy of combination therapy. Among them, the decrease in the number and quality of T cells caused by standard-dose gemcitabine may be an important factor (17). Anti-PD-1 monotherapy efficacy is limited by the number and specificity of tumor-directed T cells, and defects in either will lead to an inability to reach a critical threshold to elicit immune infiltration, especially for tumors with low mutational burdens (18). To alleviate these adverse effects on T cells, researchers have begun exploring the effect of low-dose gemcitabine in anti-tumor immunity, and investigations into the combination of low-dose gemcitabine and PD-1 for treating tumors are underway. There is some evidence that tumors exposed to low-dose gemcitabine secrete interferons that can help mature dendritic cells, which ultimately enhance T-cell responses (19). Additionally, low-dose gemcitabine selectively inhibits tumor-associated myeloid-derived suppressor cells in mice bearing 4T1 mammary carcinomas, which is beneficial to the amplification of tumor-targeting T cells (20). Moreover, a systematic review revealed that, compared with standard-dose infusion for gemcitabine, prolonged low-dose infusion is an effective and well-tolerated regimen for multiple solid tumors (21).

These data provide a strong rationale for the use of a regimen including low-dose gemcitabine along with inhibitors of the PD-1 pathway. Thus, we hypothesized that the combination of low-dose gemcitabine with PD-1 inhibitors would improve the response rate to ICI therapy and reduce the incidence of adverse reactions.

## Methods

### Research Subjects

This study retrospectively analyzed patients who received low-dose gemcitabine combined with PD-1 inhibitors in the Department of Immunotherapy, Henan Cancer Hospital, China, between January 2018 and February 2022. The follow-up was completed by 21 April 2022. Patients with advanced solid tumors that could not be resected or had metastasized were included in this study; patients were either untreated or experienced failure of standard care, predominantly for lung, hepatobiliary, pancreatic, cervical, breast, urinary carcinoma, or sarcoma. Sixty-one cases were selected based on complete baseline data (**Table 1**).

**Table 1:** Patient demographic information and baseline characteristics.

Characteristic	Patients, n (%)
Patients enrolled	61
Median age, years	60(50-65)
Sex	
Female	21(34.4)
Male	40(65.6)
Eastern Cooperative Oncology Group	
0-1	42(68.9)
≥2	19(31.1)
Type of tumor	
Lung	30(49.2)
Hepatobiliary pancreas	8(13.1)
Cervix	5(8.2)
Urinary system	6(9.8)
Sarcoma	7(11.5)
Breast	2(3.3)
Digestive tract	1(1.6)
Neck	1(1.6)
Unknown source	1(1.6)
Disease stage	
III	10(16.4)
IV	51(83.6)
Local therapy	
Previous surgery	22(36.1)
Previous radioactive therapy	27(44.3)
Lines of therapy	
1	9(14.8)
2 and more	52(85.2)
PD1 inhibitor treatment	
Initial	44(72.1)
Subsequent	17(27.9)

### Study Design and Treatment

All patients received an intravenous infusion of gemcitabine at a dose of 500 mg/m^2^ on days 1 and 8 every 3 weeks. PD-1 inhibitors were applied on the first day of gemcitabine infusion. PD-1 inhibitors included pembrolizumab, nivolumab, sintilimab, toripalimab, camrelizumab, and tislelizumab, infused once every 3 weeks at the standard dose prescribed by the physician.

### Response Assessment

Imaging examinations were conducted at baseline, and immune-related response criteria (irRC) were used every 6 weeks for response evaluation. For those who achieved a response or stable disease, study treatment was continued, and the response was evaluated using imaging every 6 weeks until disease progression or unacceptable toxicity.

### Safety Assessments

Baseline and screening assessments include medical history and a complete physical examination. Laboratory tests included a complete blood count, myocardial zymography, amylase, lipase, and thyroid-stimulating hormone level. Imaging examinations included computed tomography, magnetic resonance imaging, and positron emission tomography. Safety assessments were performed before each combination immunotherapy, namely, monitoring and recording of all AEs, routine laboratory tests, medical history, and physical examination.

### Immune Cell Assays

Whole peripheral blood samples from patients were collected and prepared using a stain-lyse-no-wash procedure to generate fluorescently-linked CD3, CD4, and CD8-labeled leukocytes using CD19, CD45, CD16, and CD56 antibodies (BD multitest 6-color TBNK reagent). Absolute lymphocyte counts and subset percentages were calculated by BD FACSCanto software. The absolute number (cells/µl) of positive cells was determined by comparing cellular events to bead events. The percentages of subsets were obtained by gating the lymphocyte populations.

### Statistical Analysis

PFS was defined as the time from the start of treatment to disease progression, death, or last follow-up. OS was defined as the time from the start of treatment to the last follow-up or death. Response assessments were displayed using waterfall plots; PFS and OS were estimated using Kaplan–Meier calculations by R 3.6.1. Multivariate analysis of PFS and OS was performed using Cox proportional hazards models in R 3.6.1. The data of immune parameters, calculated as mean ± standard deviation, were analyzed by IBM SPSS statistics 21. Student’s t-test and ANOVA were applied to determine statistically significant differences (*P <*0.05) between groups.

## Results

### Patient Characteristics

In total, there were 21 (34.4%) female and 40 (65.6%) male patients. The median patient age was 60 (50–65) years, and the median number of courses of treatment with low-dose gemcitabine combined with a PD-1 inhibitor was 3 (1–11). Forty-two patients (68.9%) had an ECOG score <2 and 19 patients (31.1%) had a score ≥2. Of those, 51 (83.6%) patients had stage IV disease; the others were stage III. Forty-nine (80.3%) had received previous local therapy, 23 had undergone surgery, and 27 had received radiation therapy. Low-dose gemcitabine combined with a PD-1 inhibitor was administered as first-line therapy in 9 patients (14.8%). A total of 44 patients (72.1%) were receiving immunotherapy for the first time, and 17 (27.9%) had received previous immunotherapy. The 30 lung tumors included 8 squamous carcinomas, 11 adenocarcinomas, 1 adenosquamous carcinoma, and 10 small-cell carcinomas. The 8 hepatobiliary pancreatic tumors included 1 hepatocellular carcinoma, 1 hepatic adenocarcinoma, 1 intrahepatic cholangiocarcinoma, 3 gallbladder cancers, and 2 pancreatic cancers. The 5 cervical tumors included 4 squamous carcinomas and 1 adenocarcinoma. The 6 urologic tumors included 1 renal adenocarcinoma, 4 urothelial carcinomas, and 1 bladder cancer. The 7 sarcomas were 2 cases on the back, 1 osteosarcoma, 1 pelvic fibrosarcoma, 1 mandibular gingival epithelioid angiosarcoma, 1 small cell synovial sarcoma of the small intestine, and 1 esophageal sarcomatoid carcinoma. Two invasive ductal breast cancer patients were molecularly classified as Luminal B: one with lung and brain metastases, and the other with liver, spleen, and bone metastases. There was one patient with esophageal squamous cell carcinoma, one patient with gingival squamous cell carcinoma, and one patient with adenocarcinoma of unknown primary. The basic information for all patients is shown in **Table 1**.

### Therapy Response

**Figure 1:**
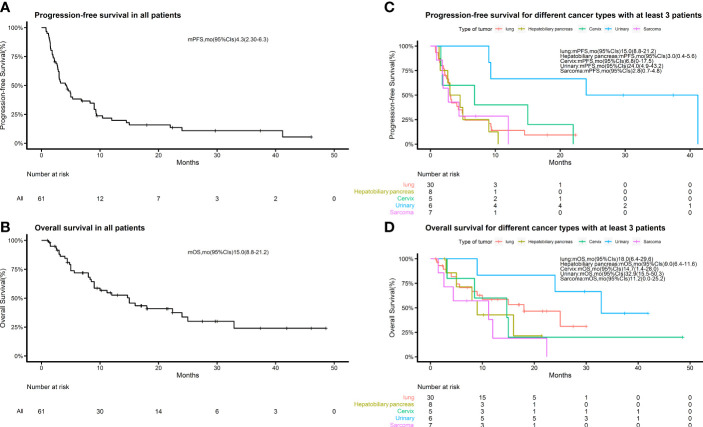
Kaplan–Meier estimates of progression-free survival and overall survival. **(A)** Kaplan–Meier estimates of progression-free survival in all patients; **(B)** Kaplan–Meier estimates of overall survival in all patients. **(C)** Kaplan–Meier estimates of progression-free survival for different cancer types with at least 3 patients in each group. **(D)** Kaplan–Meier estimates of overall survival in the groups described in **(C)**.

**Figure 2:**
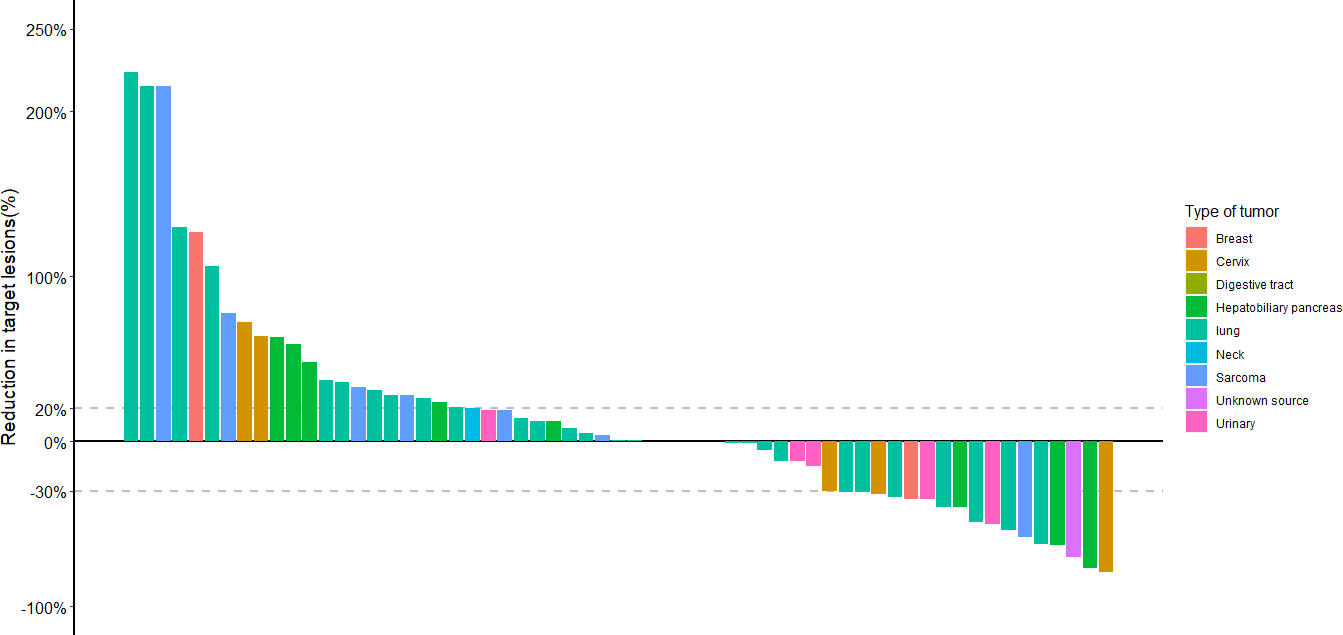
Waterfall plot showing best responses by immune-related response criteria. The best change from baseline as the sum of longest target lesion diameter per patient is shown.

We retrospectively evaluated the treatment responses of all patients. Eighteen (29.5%) achieved a partial response (PR), 20 (32.8%) had a response rated stable disease (SD), and the remaining 23 (37.7%) had progressive disease (PD), yielding an ORR of 29.5% and a disease control rate (DCR) of 62.3%. The median PFS of the 61 patients was 4.3 months (95% CI 2.3–6.3) and the median OS was 15.0 months (95% CI 8.8–21.2) (**Figure 1**). Treatment effects for patients with each cancer type are shown in **Table 2**. Among the 9 pathological types of tumors we observed, cervical and urologic tumors had the best treatment response, with 60 and 100% DCR, respectively. Two patients had invasive ductal carcinoma of the breast. One (with a response of PR) had a disease remission time of 46.1 months as of the end of follow-up, and the other (with a response of PD) had a PFS of 1.5 months and an OS of 4.4 months. One patient with esophageal squamous cell carcinoma, who was lost to follow-up, had SD without an exact survival status. One patient with gingival squamous cell carcinoma had PD, with a PFS of 1.5 months and an OS of 4.8 months. One patient with adenocarcinoma of unknown primary had a response of PR, with PFS and OS of 8.3 months, and died of cachexia. Waterfall plots showing the magnitude of change in tumor mass from baseline in response to optimal immune combination therapy in all patients are shown in **Figure 2**.

**Table 2:** Response and survival data.

Effect	Patients, n (%)
**Summary**	
Complete response	0(0)
Partial response	18(29.5)
Stable disease	20(32.8)
Progressive disease	23(37.7)
Objective response rate	18(29.5)
Disease control rate	38(62.3)
Median progression-free survival, months	4.3(2.3-6.3)
Median overall survival, months	15.0(8.8-21.2)
**Lung**	
Complete response	0(0)
Partial response	7(23.3)
Stable disease	12(40.0)
Progressive disease	11(36.7)
Objective response rate	7(23.3)
Disease control rate	19(63.3)
Median progression-free survival, months	3.0(2.6-3.4)
Median overall survival, months	18.0 (6.4-29.6)
**Hepatobiliary pancreas**	
Complete response	0(0)
Partial response	3(37.5)
Stable disease	1(12.5)
Progressive disease	4(50.0)
Objective response rate	3(37.5)
Disease control rate	4(50.0)
Median progression-free survival, months	3.0(0.4-5.6)
Median overall survival, months	9.0(6.4-11.6)
**Cervix**	
Complete response	0(0)
Partial response	3(60.0)
Stable disease	0(0)
Progressive disease	2(40.0)
Objective response rate	3(60.0)
Disease control rate	3(60.0)
Median progression-free survival, months	6.8(0-17.5)
Median overall survival, months	14.7(1.4-28.0)
**Urinary**	
Complete response	0(0)
Partial response	2(33.3)
Stable disease	4(66.7)
Progressive disease	0(0)
0bjective response rate	2(33.3)
Disease control rate	6(100)
Median progression-free survival, months	24.0(4.9-43.2)
Median overall survival, months	32.9(15.5-50.3)
**Sarcoma**		
Complete response	0(0)
Partial response	1(14.3)
Stable disease	2(28.6)
Progressive disease	4(57.1)
Objective response rate	1(14.3)
Disease control rate	3(42.9)
Median progression-free survival, months	2.8(0.7-4.8)
Median overall survival, months	11.2(0.0-25.2)
**Breast**		
Complete response	0(0)
Partial response	1(50.0)
Stable disease	0(0)
Progressive disease	1(50.0)
Objective response rate	1(50.0)
Disease control rate	1(50.0)
**Digestive tract**		
Response	Stable disease
**Neck**		
Response	Progressive disease
**Unknown source**		
Response	Partial response

### Risk Factor Analysis

**Figure 3:**
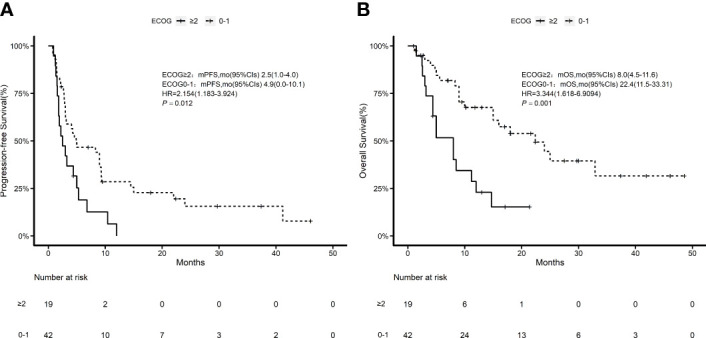
Kaplan–Meier estimates of ECOG 0–1 and ECOG ≥2 patients. **(A)** Kaplan–Meier estimates of progression-free survival; **(B)** Kaplan–Meier estimates of overall survival.

**Figure 4:**
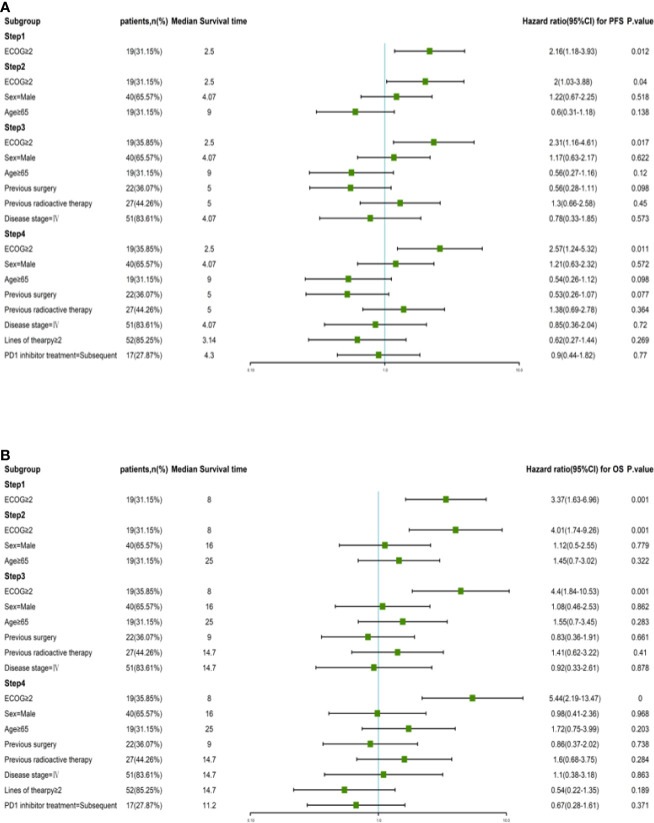
Risk factor analysis by Kaplan-Meier calculations and Cox proportional hazards models. **(A)** PFS and corresponding hazard ratios at different risk factors in all patients. **(B)** OS and corresponding hazard ratios at different risk factors in all patients.

According to the Kaplan–Meier method, we analyzed 7 risk factors for association with patient survival: gender, ECOG performance score, disease stage, previous surgical history, previous radiation therapy history, previous treatment cycle, and previous PD-1 inhibitor therapy history. In terms of outcomes, only ECOG score was a moderate influencing factor for both PFS and OS (*P* = 0.011, *P* = 0.003) among all factors (**Figure 3**). The disease stage had no statistical impact on patient survival time (**Supplementary Figure 1**). Cox multivariate regression analysis identified ECOG ≥2 as a prognostic factor in patients with advanced tumors who received this combination immunotherapy regimen for either PFS (HR = 2.57, 95% CI 1.24–5.32, P = 0.011) or OS (HR = 5.44, 95% CI 2.19–13.47, P = 0.000) (**Figure 4**).

### Immune Parameters

We analyzed the association of various pre-treatment lymphocyte subsets in peripheral blood with treatment efficacy, dividing patients into PR, SD, and PD groups. We evaluated absolute T lymphocyte count, absolute CD3^+^CD4^+^ lymphocyte count, absolute CD3^+^CD8^+^ lymphocyte count, percentage of regulatory T cells, absolute B lymphocyte count, percentage of B lymphocytes, and absolute NK cell count before treatment in both groups. No significant differences in any of these immune parameters before treatment between groups were found (*P >*0.05) (**Supplementary Figure 2**). We also examined the levels of immune cells before and after combination therapy to verify that low-dose gemcitabine did not decrease lymphocyte numbers. The results showed that the total number of T cells, B cells, and NK cells did not change significantly before and after the combination therapy was applied (**Supplementary Figure 3**).

### Safety

Treatment-related AEs are summarized in **Table 3**. The most common AE in the 61 patients was hematologic toxicity (60.7%), mainly leukopenia or thrombocytopenia. AEs occurring in ≥10% of patients were fever/chills (14.8%) and aspartate aminotransferase (AST)/alanine aminotransferase (ALT) elevation (11.4%); fever often presented as hyperpyrexia, appearing on the day of administration. Other AEs included fatigue/anorexia (6.5%), creatinine increase (6.5%), thyroid dysfunction (6.5%), hyperglycemia (3.2%), pancreatic enzyme elevation (3.3%), skin reaction (1.6%), pneumonia (1.6%), colitis (1.6%), and neurotoxicity (1.6%). A total of 17 (27.9%) patients experienced grade 3 or 4 AEs, including 4 (6.6%) who developed grade 3 leukopenia, 4 (6.6%) who developed grade 3 thrombocytopenia, 2 (3.3%) who developed serious pulmonary or abdominal infections and died, and 4 (6.6%) who presented with hyperthermia. The other 3 patients suffered from increased-grade 3 creatinine elevation, grade 3 hyperglycemia, and grade 3 pneumonia, respectively (1.6%). None of the patients developed myositis or myocarditis (**Table 3**).

**Table 3:** Treatment-related adverse events classified by grade.

Adverse event	Grade 1, n (%)	Grade 2, n (%)	Grade 3-4, n (%)
Rash/pruritus	0	1(1.6)	0
Fatigue/anorexia	1(1.6)	3(4.9)	0
Fever/chills	0	5(8.2)	4(6.6)
Infection	0	3(4.9)	2(3.3)
Haematological toxicity	11(18.0)	18 (29.5)	8(13.1)
Colitis	0	1(1.6)	0
Peripheral neuropathy	0	1(1.6)	0
Elevated amylase/lipase	2(3.3)	0	0
Elevated AST/ALT/bilirubin	6(9.8)	1(1.6)	0
Elevated creatinine	3(4.9)	0	1(1.6)
Hyperglycemia	0	1(1.6)	1(1.6)
Pneumonia	1(1.6)	0	1(1.6)
Myositis	0	0	0
Myocarditis	0	0	0
Thyroid dysfunction	1(1.6)	3(4.9)	0

## Discussion

Our study demonstrates that the use of low-dose gemcitabine along with a PD-1 inhibitor is safe and feasible in patients with advanced solid malignancies and results in an impressive 29.5% ORR and 62.3% DCR. The median PFS was 4.3 months and the median OS was 15.0 months.

Parikh et al. (22) suggested pembrolizumab along with standard dose gemcitabine as a second or third-line therapy in patients with advanced or metastatic UC had an ORR of 33%, DCR of 50%, and median PFS of 3.7 months. In the IMvigor130 study, patients with metastatic UC received first-line atezolizumab plus platinum chemotherapy (1,000 mg/m^2^), gemcitabine plus carboplatin or cisplatin, and obtained a median PFS of 8.2 months and a median OS of 16.0 months (23). In our study, ORR and DCR in patients with urothelial carcinoma were 33 and 100%, respectively, and the median PFS and OS were 24.0 and 32.9 months, respectively. These data suggest that a higher response rate may be achieved with low-dose, rather than standard-dose, gemcitabine plus a PD1 inhibitor. For cervical cancer, the phase II NCT02257528/NRG-GY002 trial evaluated the efficacy and safety of nivolumab in 26 persistent or recurrent patients. In that trial, the ORR was only 4%, and the estimated PFS and OS at 6.0 months were 16 and 78.4%, respectively (24). In our study, ORR in patients with cervical cancer was 60%; median PFS and OS were 6.8 and 14.7 months, respectively; these results suggest that the efficacy of ICIs combined with chemotherapy is much better than that of single-agent ICIs.

Different doses of gemcitabine have different effects on peripheral lymphocytes. In a mouse model, gemcitabine at high doses resulted in complete reductions in CD4^+^, CD8^+^, and B220^+^ cell numbers (25), whereas low doses selectively reduced myeloid-derived suppressor cell levels without affecting lymphocyte counts (20). In a clinical setting, gemcitabine caused statistically significant decreases in the absolute numbers of CD3 and CD20 lymphocytes when administered in routine doses (26). Unlike in other studies, the chemotherapy drug gemcitabine used in our study was administered at a lower dose; the results showed that this dose did not affect the absolute numbers of T, B, and NK cells (**Supplementary Figure 3**). The regimen proposed here does not reduce the number of lymphocytes; ensuring sufficient immune cells may be an important reason why the combined regimen can exert a superior therapeutic effect.

In our previous study, we found that elevated B-cell levels in the peripheral blood were associated with poor prognosis of anti-PD-1-based immunotherapy, which indicates that B cells may affect the efficacy of immunotherapy with PD-1 inhibitors (27). To evaluate whether there is a correlation between B cells and the efficacy of combination therapy, we detected pre-treatment levels of various lymphocytes, including B cells, in patients within the PR, SD, and PR groups; however, no significant differences were observed (**Supplementary Figure 2**), suggesting that in addition to B cells, other factors can also affect the efficacy of immunotherapy.

Our study has several limitations: first, the retrospective design may have permitted selection bias; second, the non-interventional design may have led to heterogeneity in patient management and poor data quality; third, the heterogeneity of tumor types and small numbers for each type of tumor limits the conclusions that can be drawn for a given tumor type (but, the larger sample size was valuable for stratified analysis to identify the efficiency of combination therapy, especially for cervical and urologic cancers); fourth, data related to the positive predictive biomarkers for PD-1 inhibitor therapy were not presented because of incomplete baseline data. To address these limitations, we are currently working on a prospective clinical trial on a fixed tumor (NCT04331626) to confirm these findings.

## Conclusion

Low-dose gemcitabine combined with PD-1 inhibitors offers a novel option for treatment in patients with advanced malignancies and appears particularly promising for post-operative patients. Our results suggest that patients with urological and cervical cancers benefit significantly and that this specific combination therapy is well tolerated. Future studies with larger sample sizes will help further verify these results.

## Data Availability Statement

The original contributions presented in the study are included in the article/**Supplementary Material**. Further inquiries can be directed to the corresponding authors.

## Ethics Statement

The studies involving human participants were reviewed and approved by Medical Ethics Committee of Henan caner hospital (2019090507). The patients/participants provided their written informed consent to participate in this study.

## Author Contributions

HH, LP, and BZ performed experiments, analyzed data, and wrote the manuscript. BZ and BT edited the manuscript. HH, YY, XZ, LZ, XF, TL, LH, and PQ were responsible for patient management. LC, XY, YL, WW, ZY, LR, and LG analyzed data. LH, QG, and ZW designed and supervised the study. All authors contributed to the article and approved the submitted version.

## Funding

This work was supported by the National Natural Science Foundation of China (Grant No. 81972690) and the Medical Science and Technology Research Project of Health Commission of Henan Province (YXKC2021007).The funding bodies played no role in the design of the study and collection, analysis, or in the interpretation of data and writing the manuscript.

## Conflict of Interest

The authors declare that the research was conducted in the absence of any commercial or financial relationships that could be construed as a potential conflict of interest.

## Publisher’s Note

All claims expressed in this article are solely those of the authors and do not necessarily represent those of their affiliated organizations, or those of the publisher, the editors and the reviewers. Any product that may be evaluated in this article, or claim that may be made by its manufacturer, is not guaranteed or endorsed by the publisher.

## Supplementary Material

Supplementary Figure 1 Kaplan–Meier estimates of III and IV disease stage patients. **(A)** Kaplan–Meier estimates of progression-free survival; **(B)** Kaplan–Meier estimates of overall survival.

Supplementary Figure 2 >Immune parameters in PR, SD and PD group. The baseline peripheral blood absolute T lymphocyte count **(A)**, absolute CD3^+^CD4^+^ lymphocyte count **(B)**, absolute CD3^+^CD8^+^ lymphocyte count **(C)**, percentage of regulatory T cells **(D)**, absolute B lymphocyte count **(E)**, percentage of B lymphocytes **(F)**, absolute NK cell count **(G)**, and percentage of NK cells **(H)** were compared in each two groups. The data calculated as the means ± standard deviation using IBM SPSS statistics 21, no significant difference in any pre-treatment immune parameters were observed between groups (P > 0.05).

Supplementary Figure 3 Lymphocyte levels before and after low-dose gemcitabine combined with PD-1 inhibitor treatment. Shown are the total number of T **(A)**, B **(B)** and NK **(C)** cells in peripheral blood at the baseline, after 1st cycle treatment and 2nd cycle treatment.
