# Supplementary figures and images for: Combination of Low-Dose Gemcitabine and PD-1 Inhibitors for Treatment in Patients With Advanced Malignancies
Source: Front Immunol. 2022 Jul 13;13:882172. doi: 10.3389/fimmu.2022.882172 (PMC9328170; doi:10.3389/fimmu.2022.882172)

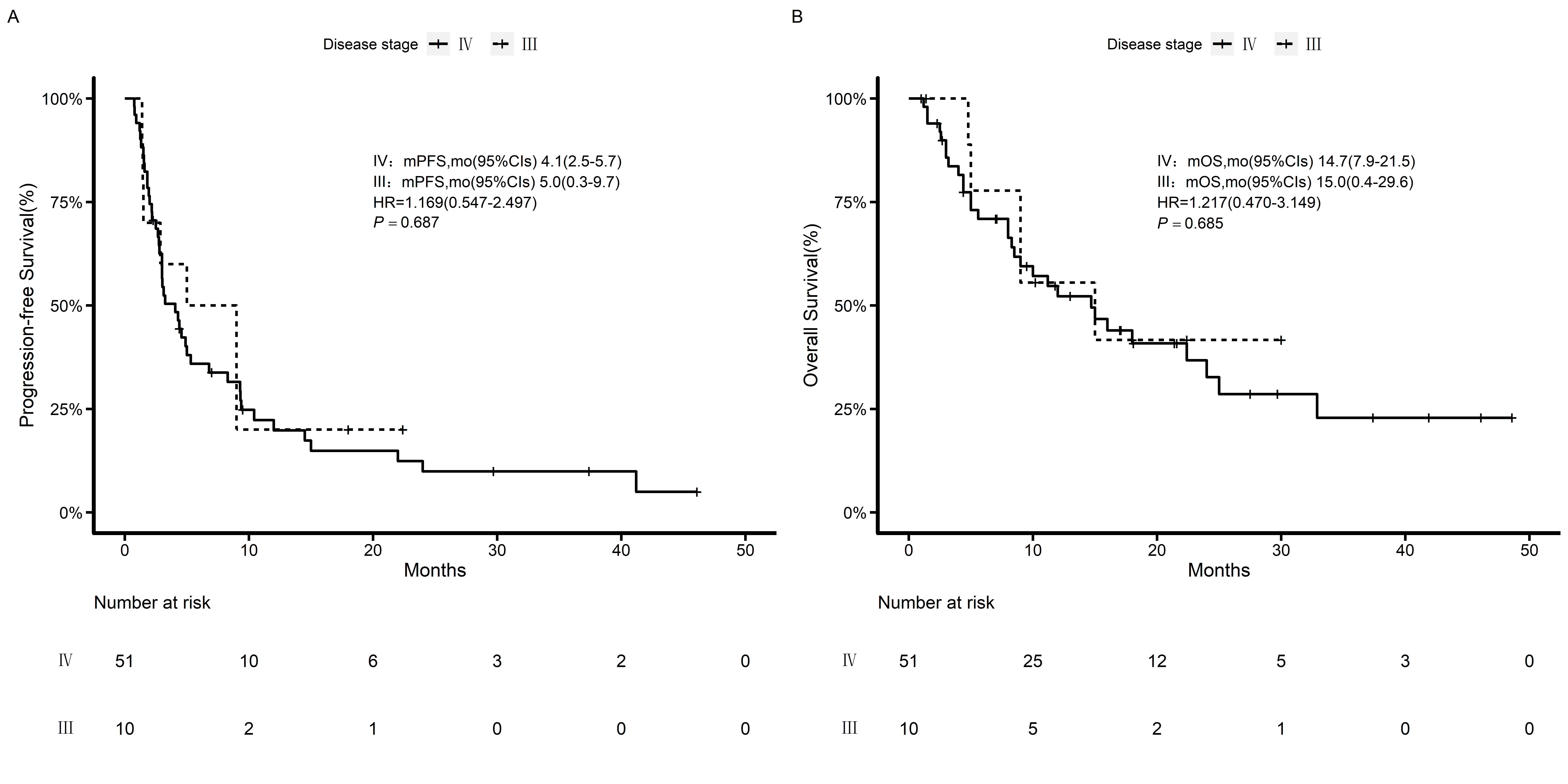

Supplement: Supplementary Figure 1 — Kaplan–Meier estimates of III and IV disease stage patients. (A) Kaplan–Meier estimates of progression-free survival; (B) Kaplan–Meier estimates of overall survival. [file Image_1.tiff]

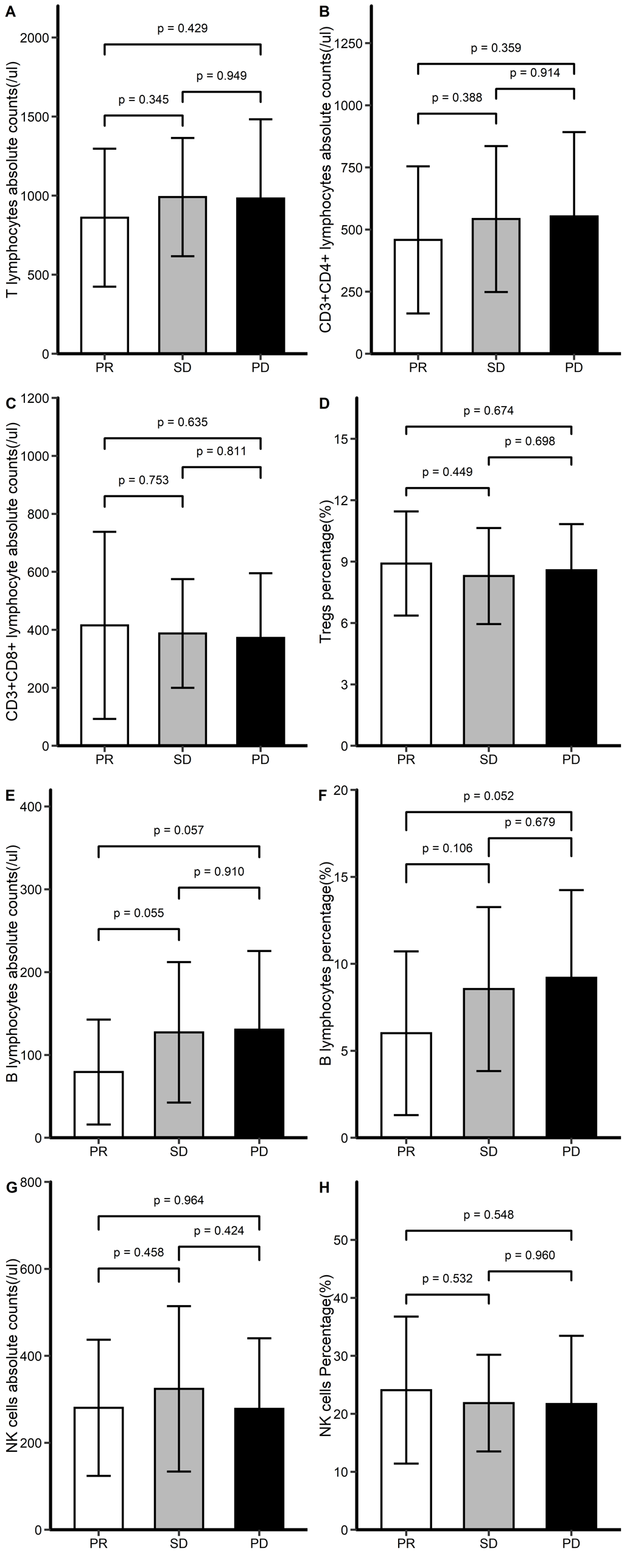

Supplement: Supplementary Figure 2 — >Immune parameters in PR, SD and PD group. The baseline peripheral blood absolute T lymphocyte count (A), absolute CD3+CD4+ lymphocyte count (B), absolute CD3+CD8+ lymphocyte count (C), percentage of regulatory T cells (D), absolute B lymphocyte count (E), percentage of B lymphocytes (F), absolute NK cell count (G), and percentage of NK cells (H) were compared in each two groups. The data calculated as the means ± standard deviation using IBM SPSS statistics 21, no significant difference in any pre-treatment immune parameters were observed between groups (P > 0.05). [file Image_2.tiff]

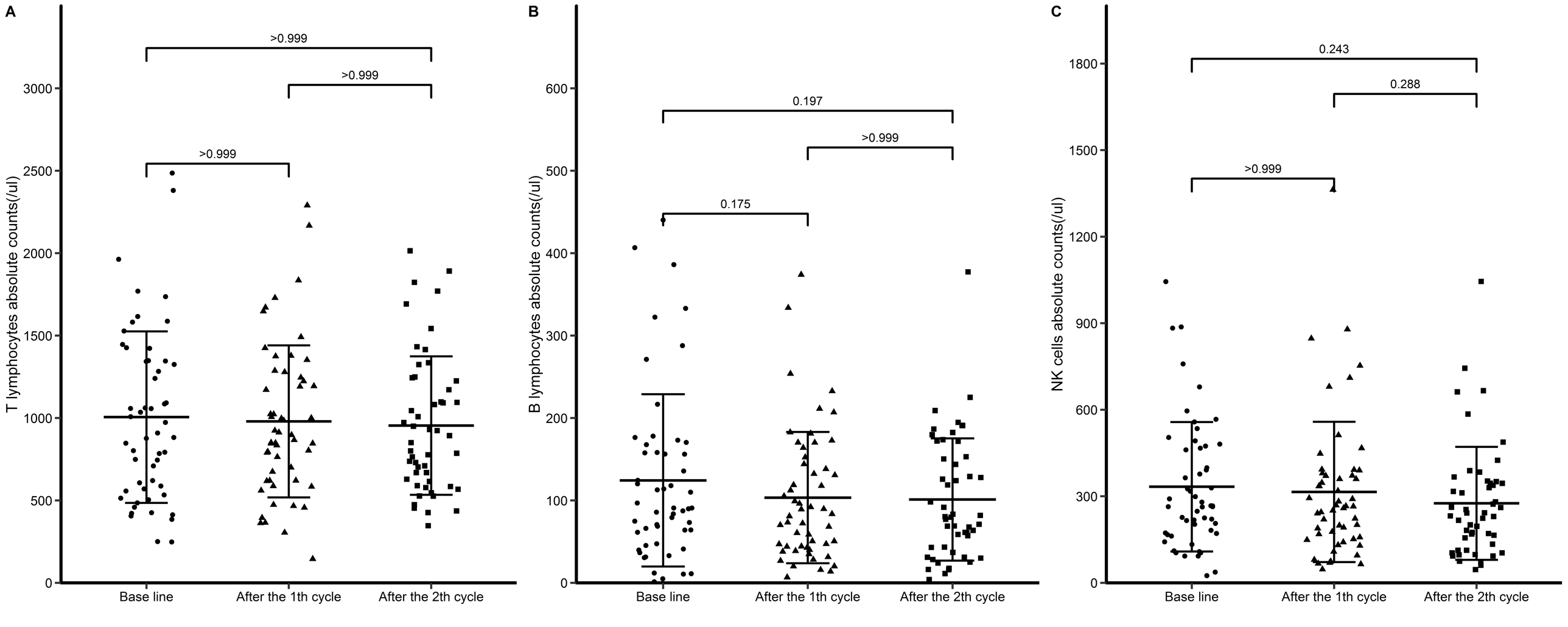

Supplement: Supplementary Figure 3 — Lymphocyte levels before and after low-dose gemcitabine combined with PD-1 inhibitor treatment. Shown are the total number of T (A), B (B) and NK (C) cells in peripheral blood at the baseline, after 1st cycle treatment and 2nd cycle treatment. [file Image_3.tiff]
